# Chronic administration of LIMK2 inhibitors alleviates cavernosal veno-occlusive dysfunction through suppression of cavernosal fibrosis in a rat model of erectile dysfunction after cavernosal nerve injury

**DOI:** 10.1371/journal.pone.0213586

**Published:** 2019-03-14

**Authors:** Juhyun Park, Hwancheol Son, Ji Sun Chai, Soo Woong Kim, Jae-Seung Paick, Min Chul Cho

**Affiliations:** 1 Department of Urology, Seoul National University College of Medicine, Boramae Medical Center, Seoul, Korea; 2 Department of Urology, Seoul National University College of Medicine, Seoul National University Hospital, Seoul, Korea; California Health Sciences University, UNITED STATES

## Abstract

We evaluated whether chronic administration of LIMK2-inhibitors could improve erectile function by alleviating CVOD through suppressing cavernosal fibrosis in a rat model of cavernosal nerve crush-injury (CNCI). Forty-two 12-week-old rats were equally categorized into the three groups: sham-surgery (S), CNCI (I), and CNCI treated with LIMK2-inhibitors (L). The L-group was treated with daily intraperitoneal injection of LIMK2-inhibitors (10.0 mg/kg) for 30-days after surgery. Erectile function was assessed using dynamic-infusion-cavernosometry (DIC). Penile tissue was processed for Masson’s-trichrome staining, Western-blotting, and double immunofluorescence. The I-group showed significantly higher maintenance and drop rates as well as lower papaverine response, compared to the S-group. Chronic inhibition of LIMK2 in the L-group significantly improved the DIC parameters compared to those in the I-group, although the parameters were not completely restored to normal control values. Also, the I-group showed a reduced smooth muscle (SM)-to-collagen ratio, decreased immunohistochemical staining for α-SM-actin, increased number of fibroblasts positive for phosphorylated Cofilin, increased LIMK2/Cofilin phosphorylation and increased protein expression of Collagen-1 or Fibronectin, compared to the S-group. The L-group showed significant improvements in SM/collagen ratio and the deposition of Collagen-1 or Fibronectin compared to the I-group, although not completely normalized. According to the densitometry and confocal microscopy results, the L-group showed restoration of LIMK2/Cofilin phosphorylation and amount of fibroblasts positive for phosphorylated Cofilin to the normal control value. In conclusion, chronic inhibition of LIMK2 can improve CVOD and ED by alleviating cavernosal fibrosis via normalizing the LIMK2/Cofilin pathway.

## Introduction

Despite technical refinements in nerve-sparing radical prostatectomy (ns-RP), a significant proportion of men still suffer from erectile dysfunction (ED) as a major complication of RP [1–6]. Even when surgically meticulous techniques are applied to avoid direct damage to the cavernosal nerve (CN), ED can occur as a consequence of neuropraxia caused by traction, compression, coagulation and minimal manipulation [4, 7]. The neuropraxia induces loss of nocturnal penile tumescence, and subsequently, low oxygen supply to the penis during the early postoperative period [7]. This penile hypoxia leads to irreversible structural changes such as cavernosal apoptosis and fibrosis, thereby resulting in cavernosal veno-occlusive dysfunction (CVOD), which is known as the key pathophysiology of post-RP ED [7–10].

Although cellular dysfunction and organ failure ultimately ensue from the progression of fibrosis [11], there is scarcity of data on molecular mechanisms leading to cavernosal fibrosis. Up to date, several previous studies have reported that activated RhoA/ROCK1 or ROCK2 pathway plays a critical role in the development or progression of vascular fibrosis in cardiovascular diseases [12–16]. In this context, we showed that the RhoA/ROCK1/LIM-kinase 2 (LIMK2)/Cofilin pathway contributed to cavernosal fibrosis with a loss of smooth muscle (SM) after CN injuries [17, 18]. In addition, early inhibition of ROCK, an upstream molecule of LIMK2 in the ROCK1/LIMK2/Cofilin pathway could prevent both corporal apoptosis and fibrosis after CN injury by suppressing the Akt-driven and ROCK1/LIMK2/Cofilin pathways, preventing CVOD and ED [18]. Furthermore, recent studies showed that activated RhoA/ROCK2 pathway played a critical role in the development of penile structural alterations and ED in a rat model of CN injury [19, 20]. However, considering the risk for significant side effects from systemic use of ROCK inhibitors, we paid attention to a LIMK2 inhibitor, a down-stream target of ROCK, in order to identify a reasonable strategy for the treatment of cavernosal fibrosis after CN injury. Selectively inhibiting a downstream pathway of ROCK such as LIMK2/Cofilin might be better than targeting ROCK itself in terms of both efficacy and safety [21].

Recently, we demonstrated that inhibition of LIMK2 during the short-term period beginning from the immediate post-injury period improved cavernosal fibrosis and erectile response to electrostimulation by normalizing the LIMK2/Cofilin pathway in a rat model of CN crush injury (CNCI) [22]. Thus, the possibility of targeting LIMK2, a key down-stream effector of the TGF-β/ROCK1/LIMK2/Cofilin pathway, for alleviation of cavernosal fibrosis caused by CN injury was suggested.

However, whether chronic administration of LIMK2 inhibitors can improve cavernosal veno-occlusive function (CVOF) through suppression of cavernosal fibrosis remains to be determined, because CVOD is known as a major contributor to post-RP ED. Thus, the aim of this study was to determine whether chronic administration of LIMK2 inhibitors could alleviate ED by improving CVOF through suppression of cavernosal fibrosis in a rat model of CNCI, to contribute to elucidation of the role of LIMK2 inhibition in improvement of CVOD, the major pathophysiologic mechanism of post-RP ED.

## Materials and methods

### Experimental design

Forty-two 12-week-old rats, weighing 325–365 g, were equally divided into the following three experimental groups (n = 14 each): sham surgery (S), bilateral CNCI (I), and CNCI treated with LIMK2 inhibitors (L). All rats in the L group received daily intraperitoneal injection of 10.0 mg/kg LX-7101 (Cellagen-Technology, USA) for 30 days beginning from the following day after surgery [22]. The rats in the S and I groups received daily intraperitoneal administration of saline vehicle only. Rats were euthanized in the CO_**2**_ chamber when they were moribund or had clinical signs such as dyspnea, cyanosis, low temperature, or paralysis of one or more extremities. Regarding the surgical procedures, the rats were anesthetized using intraperitoneal injections of zoletil (10 mg/kg) and isoflurane inhalation. In the S group, pelvic dissection was performed to identify both CNs without causing direct damage to the CNs. In the I group, which approximated the clinical situation in men undergoing ns-RP, two 80-second compressions of both CNs were performed 3–4 mm distal to the major pelvic ganglion using a microsurgical vascular clamp. After a washout period of 48 hours (treatment discontinuance), erectile function was assessed at 30 days after surgery. The protocol was approved by the Institutional Animal Care and Use Committee of the Clinical Research Institute at our hospital, the Association-for-Assessment-and-Accreditation-of-Laboratory-Animal-Care (AAALAC)-accredited facility.

### In vivo assessment of CVOF and sample procurement

We evaluated CVOF in anesthetized rats using dynamic infusion cavernosometry (DIC) at 30 days after surgery, as described previously [23]. Briefly, after rats were anesthetized with zoletil (50 mg/kg), a 24-gauge angiocatheter was then introduced into the carotid artery and connected to a pressure channel for continuous monitoring of mean arterial pressure (MAP). A 26-gauge needle connected to a pressure transducer was introduced into the corpus cavernosum, and baseline intracavernous pressure (ICP) was recorded. The contralateral cavernosum was cannulated using another 26-gauge needle. Then, 0.1 ml papaverine (10 mg/ml) was administered through the infusion cannula into the cavernosum. The ICP during tumescence induced by papaverine infusion (5-min after the infusion) was recorded as ICP after papaverine administration. Saline was then infused through the cannula, starting at a rate of 0.05 ml/min. The infusion rate was increased by 0.05 ml/min every 10-sec until the ICP reached a plateau pressure (80 mmHg). The infusion rate required to maintain the ICP at 80 mmHg was defined as the maintenance rate. After the infusion was stopped, the fall in the ICP over the subsequent 1-min was recorded as the drop rate. After the CVOF was determined, all rats were euthanized by open thoracotomy. Then, we harvested the whole penis from each rat. The middle part of the skin-denuded penile shaft was fixed overnight in 10% formaldehyde solution and embedded in paraffin-wax for histological studies. The remaining penile tissues were frozen in liquid nitrogen and stored at -80°C until processing. The rats were carefully monitored and there were no unexpected deaths during the experiment. By the appropriate use of anesthetics, we minimized the distress of animals before any potentially stressful or painful procedures.

### Masson’s trichrome staining

Masson’s trichrome staining was performed according to a standard protocol, as described previously [17, 18, 22, 23]. Seven rats from each group (two sections per rat) were analyzed. We analyzed ×40 magnification images of the penis comprising one-half of the corpora cavernosa, using the Image-Pro-Plus 4.5 software (Medica-Cybernetics, Silver Spring, MD, USA). In each stained slide, the SM (stained in red)/collagen (stained in blue) ratio was determined. The slides were evaluated by an independent observer who was blinded to group allocation.

### Immunohistochemical staining for protein expression of alpha-SM actin

Immunohistochemical staining for alpha-SM actin (α-SMA) was performed to assess the percentage of the SM cell component (% α-SMA) using a primary antibody against α-SMA (1:100; Dako, Denmark), as described in previous investigations [17, 18, 22, 23]. Seven rats from each group (two sections per rat) were analyzed. In each stained slide, we performed quantitative image analyses of × 40 magnification images, using Image-Pro-Plus 4.5 software. We analyzed ×40 magnification images of the penis comprising one-half of the corpora cavernosa, and % α-SMA in the total area of one-half of the cavernosa was measured.

### Double immunofluorescent staining and confocal laser microscopy

To assess the content of fibroblasts positive for phosphorylated Cofilin (co-localization of vimentin with phosphorylated Cofilin) and to evaluate the efficacy of LIMK2 inhibition in improving CVOF through the decreased content of cavernosal fibroblasts positive for phosphorylated Cofilin, double immunofluorescence microscopy was carried out using paraffin-embedded sections (2.5 μm) of penile tissues, as previously described [22, 23]. Seven rats from each group (two sections per rat) were analyzed. The sections were incubated with primary antibodies to phosphorylated Cofilin (1:50, AbCam, UK) and Vimentin (1:100, Dako), a fibroblast marker. Following several washes in phosphate-buffered saline, the sections were incubated with two secondary antibodies (goat anti-rabbit IgG-594 and goat anti-mouse IgG-488) in 1% BSA for 1 hour at room temperature. We obtained digital images by a confocal microscope (Leica-TCS-SP8, Leica-Microsystems, Germany). Under confocal microscopy, the number of fibroblasts positive for phosphorylated Cofilin (yellow) was quantified from five randomly selected high power fields (white arrow).

### Western-blot analysis

Western-blot analysis was performed as previously described [17, 18, 22, 23]. The following primary antibodies were used: anti-ROCK1 (1:2,000, Cell-Signaling-Technology, USA), anti-phospho-LIMK2 (phospho-T505) (1:1,000, Abcam), anti-LIMK2 (1:2,000, Abcam), anti-phospho-Cofilin (1:500, Abcam), anti-Cofilin (1:1,000, Abcam), anti-Collagen-1 (1:2,000, Abcam) and anti-Fibronectin (1:2,000, Abcam). Results were quantified by densitometry and normalized to β-actin expression.

### Statistical analysis

All data were reported as means ± standard errors of the mean (SEM). Statistical comparisons among groups were performed using the Mann-Whitney-U-test. The reported p-values were two-sided. A p < 0.05 was considered statistically significant. The Statistical Package for the Social Sciences, Version 20.0 (IBM SPSS Statistics for Windows, Armonk, NY: IBM-Corp.) was used for all analyses.

## Results

### Effect of chronically administered LIMK2 inhibitors on CVOF in a rat model of bilateral CNCI

**Fig 1:**
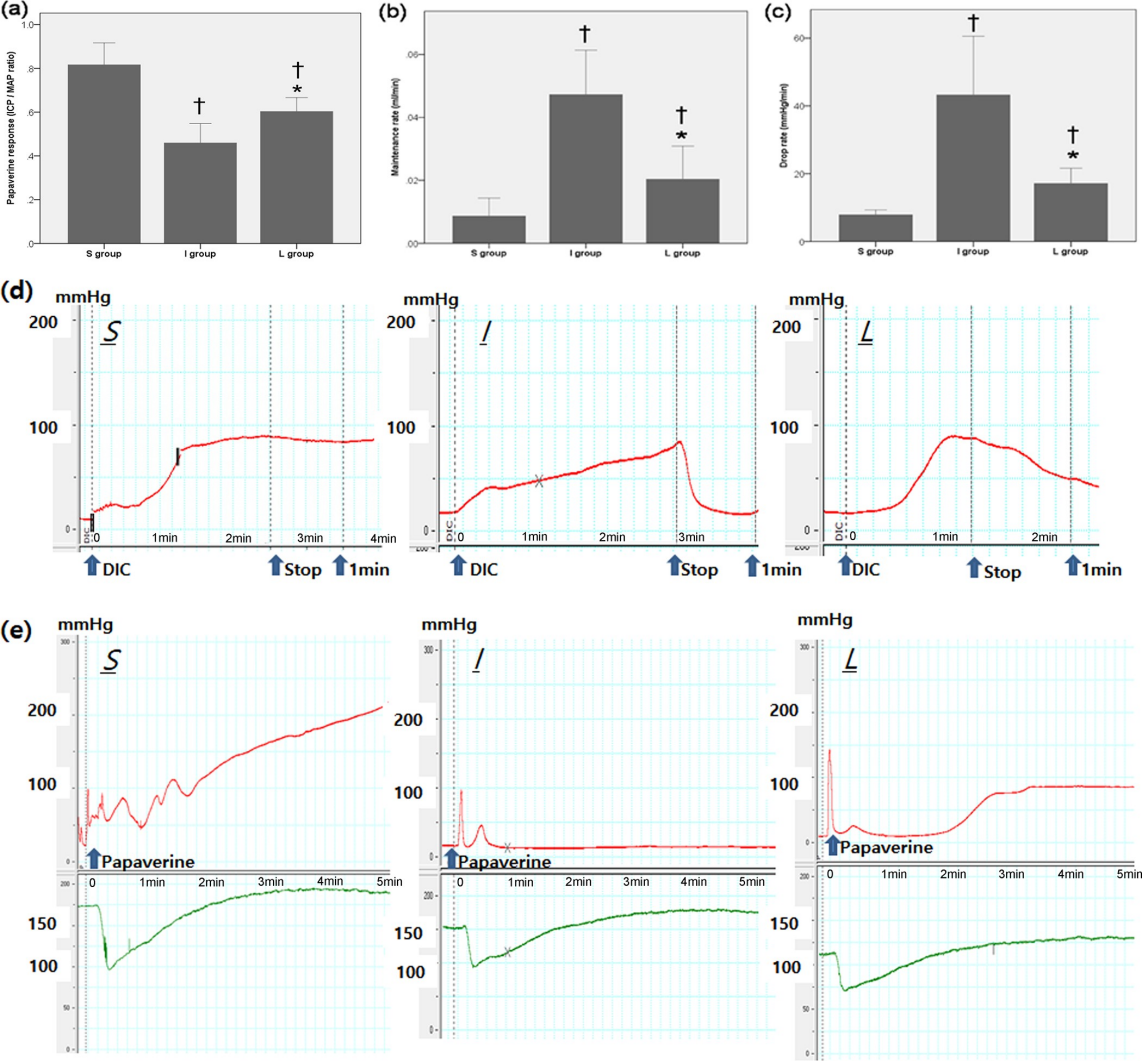
Bar graphs and representative traces showing the comparison in CVOF at 30 days after surgery among the S, I and L groups. (a) Papaverine response, (b) Maintenance rate, (c) Drop rate, (d) Representative trace of dynamic infusion cavernosometry (DIC), and (e) Representative trace of papaverine-induced intracavernous pressure (ICP) / mean arterial pressure (MAP), CVOF = cavernosal veno-occlusive function, CNCI = cavernous nerve crush injury, S = sham surgery group, I = bilateral CNCI group, L = bilateral CNCI group treated with chronic administration of LIMK2 inhibitors. * p < 0.05 vs. I group; † p < 0.05 vs. S group.

There was no significant difference in the MAP or body weight change among the three groups. The mean MAP (± SEM) in each group was 127.8 ± 6.3 mmHg in the S group, 118.1 ± 6.4 mmHg in the I group and 123.9 ± 5.6 mmHg in the L group, respectively. To determine CVOF and to demonstrate the physiological relevance of chronic administration of LIMK inhibitors, DIC was performed. The I group had significantly higher maintenance rates, higher drop rates and lower papaverine response, compared to the S group (Fig 1). Daily administration of LIMK2 inhibitors for 30 days in the L group significantly alleviated the DIC parameters compared to those in the I group, although the parameters in the L group were not completely restored to the values observed in the S group (Fig 1).

### Effect of chronic administration of LIMK2 inhibitors on cavernosal fibrosis and the LIMK2/Cofilin pathway in a rat model of bilateral CNCI

**Fig 2:**
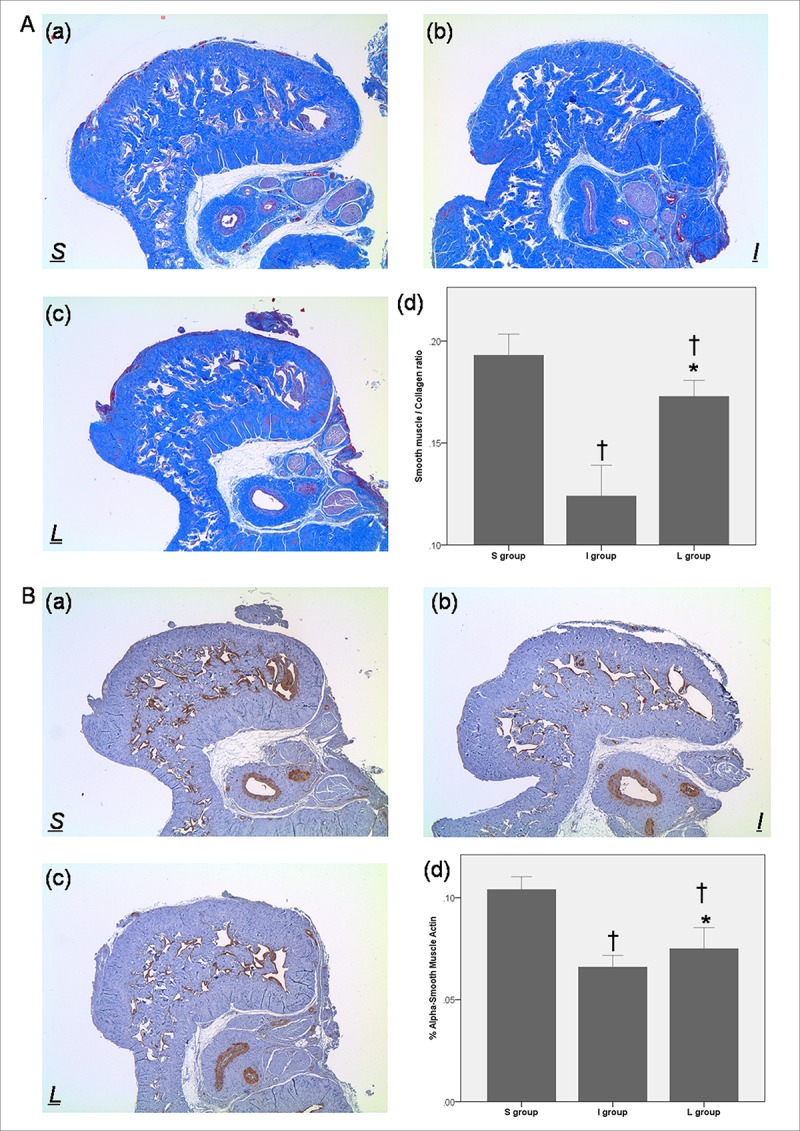
Effect of chronic administration of LIMK2 inhibitors on smooth muscle/collagen ratio and smooth muscle content after 30 days of surgery. (A) Representative images for Masson’s trichrome staining in (a) S group, (b) I group, and (c) L group. Smooth muscle and collagen fibers were stained in red and blue, respectively (magnification ×40). (d) A bar graph showing the comparison of the smooth muscle/collagen ratio (mean ± SEM) among the S, I and L groups. (B) Representative images for immunohistochemical staining of α-SMA in (a) S group, (b) I group, and (c) L group. (d) A bar graph showing the comparison of smooth muscle content (mean ± SEM) between the S and I groups. Data represent the percentage of smooth muscle fibers in a given area. S = sham surgery group, I = bilateral CNCI group, L = bilateral CNCI group treated with chronic administration of LIMK2 inhibitors. * p < 0.05 vs. I group; † p < 0.05 vs. S group.

**Fig 3:**
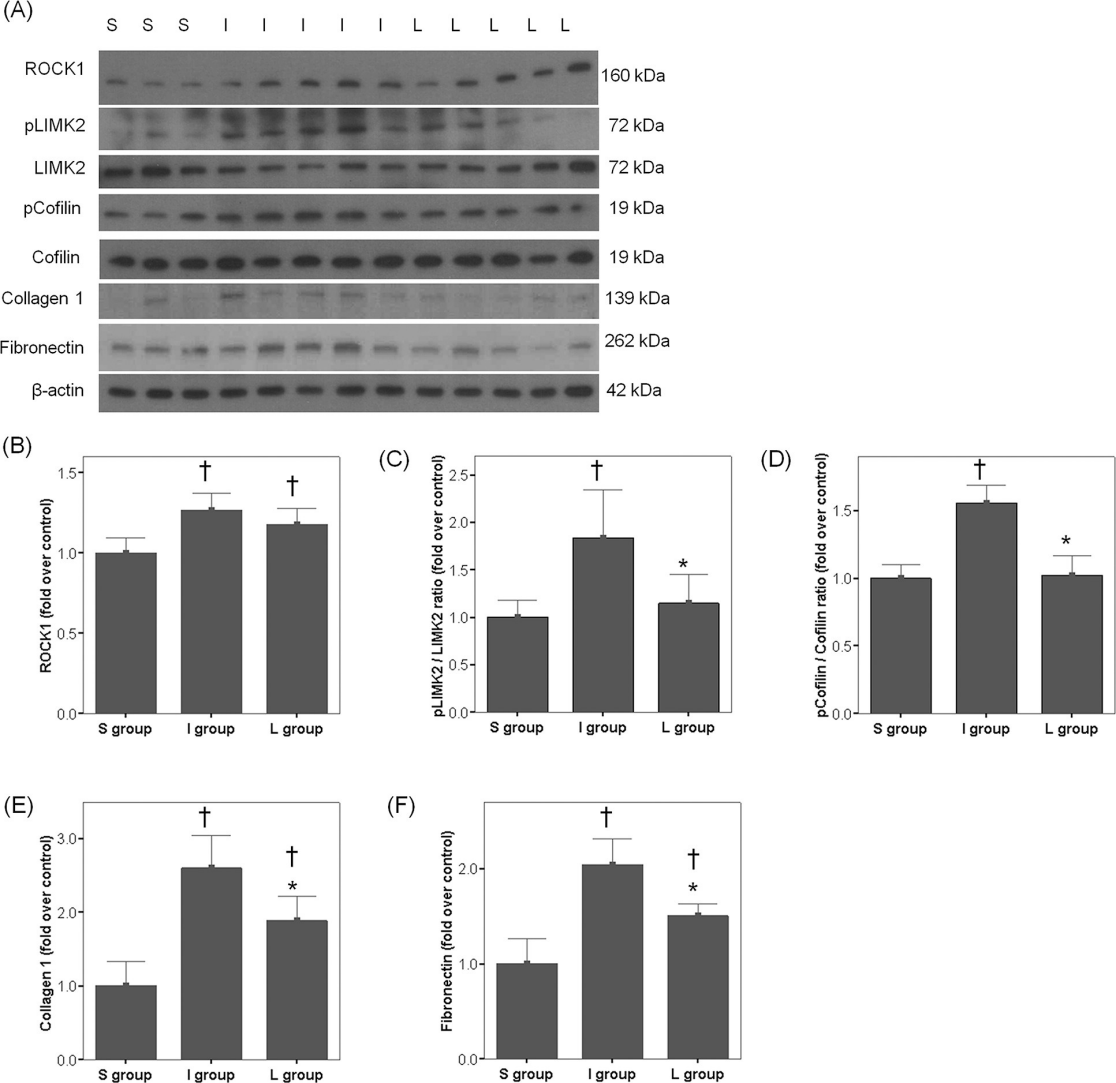
(A) Representative western blot images and bar graphs (mean ± SEM) showing the comparison in the protein expression of (B) ROCK1, (C) phosphorylated LIMK2/total LIMK2, (D) phosphorylated Cofilin/total Cofilin, (E) Collagen 1, and (F) Fibronectin in the cavernosal tissues among the S, I and L groups using densitometry. The results were normalized by β-actin expression and presented as fold changes over controls. S = sham surgery group, I = bilateral CNCI group, L = bilateral CNCI group treated with chronic administration of LIMK2 inhibitors. * p < 0.05 vs. I group; † p < 0.05 vs. S group.

The I group had a significantly decreased SM/collagen ratio, reduced immunohistochemical staining for α-SMA and increased protein expression of Collagen-1 or Fibronectin in the cavernosal tissue, compared to the S group (Figs 2 and 3). Daily administration of LIMK2-inhibitors for 30 days in the L group significantly improved the SM/collagen ratio and protein expression of Collagen-1 or Fibronectin, although the ratio was not completely restored to the normal value (Figs 2 and 3). Also, the content of α-SMA in the L group was slightly increased compared with that in the I group, but the increment was only modest (Figs 2 and 3).

**Fig 4:**
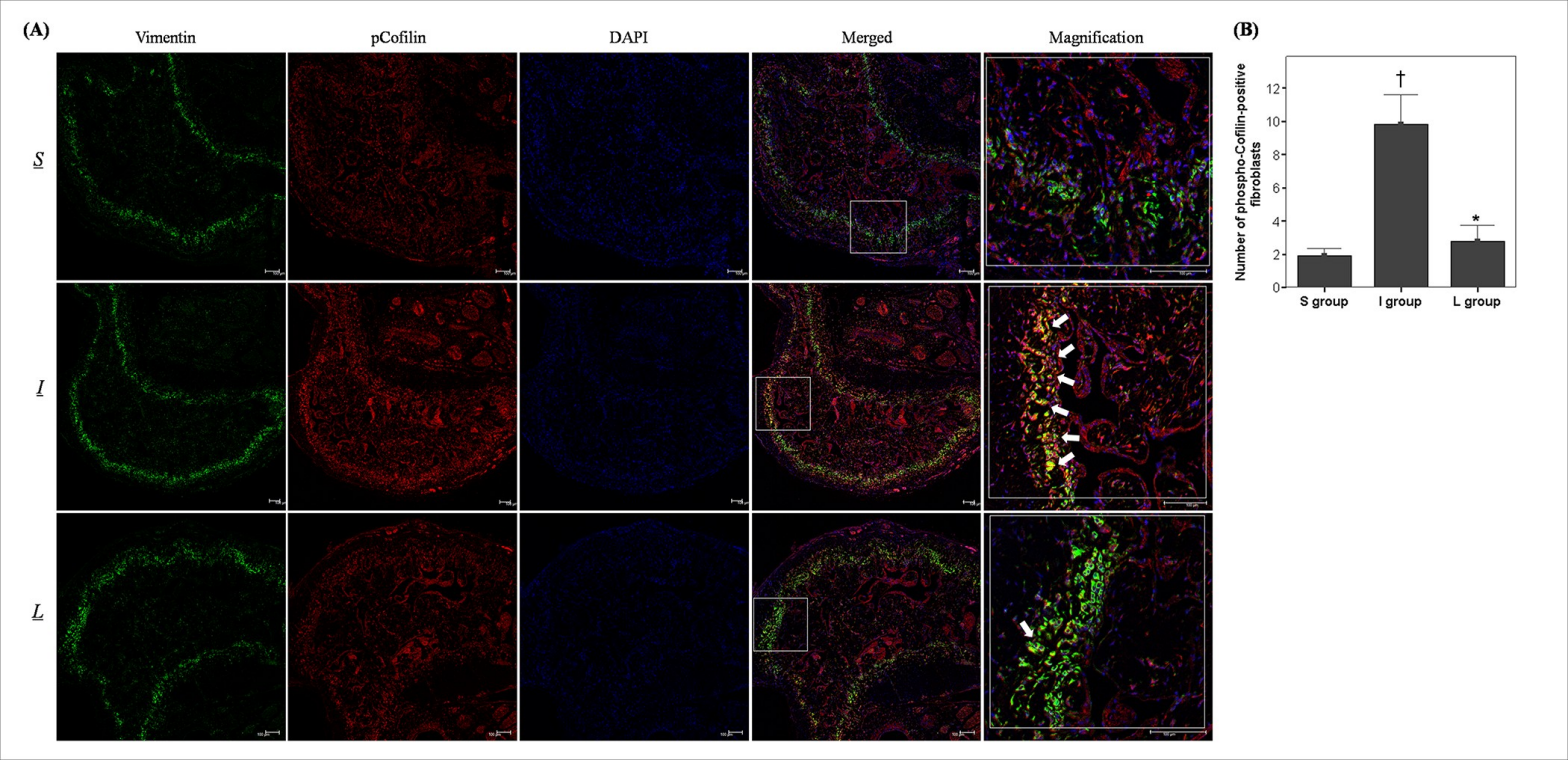
Effect of chronic administration of LIMK2 inhibitors on the content of fibroblasts positive for phosphorylated Cofilin after 30 days of surgery. (A) Representative images for double immunofluorescent staining of cavernosal tissue with anti-Vimentin and anti-phospho-Cofilin in the S, I and L groups using confocal microscope. White arrows indicate significant expression of phosphorylated Cofilin in cavernosal fibroblasts (yellow color in merged or magnified image). Scale bar = 100 μm. (B) A bar graph showing the comparison in fibroblasts positive for phosphorylated Cofilin (mean ± SEM) among the S, I and L groups. S = sham surgery group, I = bilateral CNCI group, L = bilateral CNCI group treated with chronic administration of LIMK2 inhibitors. * p < 0.05 vs. I group; † p < 0.05 vs. S group.

According to the double immunofluorescent staining of cavernous tissue with antibodies to phosphorylated Cofilin and Vimentin (Fig 4), the fibroblasts positive for phosphorylated Cofilin (co-localization of vimentin with phosphorylated Cofilin) were distributed mainly in the subtunical area. Also, the number of fibroblasts positive for phosphorylated Cofilin in the I group was significantly increased compared to that in the S group. Daily administration of LIMK2 inhibitors for 30 days in the L group restored this number to the value observed in the S group (Fig 4).

The densitometry analysis showed that LIMK2 phosphorylation and Cofilin phosphorylation in the I group were significantly higher than those in the S group (Fig 3). Daily administration of LIMK2 inhibitors for 30 days in the L group restored the dysregulated LIMK2/Cofilin pathway (Fig 3). Chronic inhibition of LIMK2 did not affect protein expression of ROCK1.

## Discussion

Damage to CNs during RP is a factor that causes postoperative cavernosal fibrosis [4, 7, 8]. Cavernosal fibrosis with loss of SM induced by CN injuries predisposes to the development of CVOD after RP [7, 24]. Thus, alleviation of CVOD through suppression of cavernosal fibrosis can be a meaningful strategy for improving post-RP ED. According to our recent study using LIMK2 inhibitors, the LIMK2/Cofilin pathway may play a critical role in the development of cavernosal fibrosis caused by CNCI, suggesting a therapeutic strategy targeting the LIMK2/Cofilin pathway for alleviation of cavernosal fibrosis and post-RP ED [22]. In this context, the present study was designed to identify the beneficial effect of chronic treatment with LIMK2 inhibitors on CVOD caused by CNCI. This study demonstrated that chronic treatment with LIMK2 inhibitors alleviated cavernosal fibrosis through normalization of the LIMK2/Cofilin pathway, thereby resulting in improvement of CVOD, the main pathophysiologic mechanism of post-RP ED.

The present study demonstrated that cavernosal fibrosis regulated by the ROCK1/LIMK2/Cofilin pathway could play a critical role in the development of CVOD caused by CN injuries during RP. A previous study by Ferrini et al. identified time course on the development of cavernosal fibrosis and CVOD after CN injury, and demonstrated that cavernosal fibrosis developed starting from the early post-injury period and was progressive over time, suggesting CVOD as the major pathophysiology of post-RP ED [24]. In this context, a few previous studies have shown that daily administration of type-5 phosphodiesterase inhibitors (PDE5Is) or nitric oxide donor improved CVOD through alleviation of histological alterations such as cavernosal fibrosis after CN injuries [25–28]. Meanwhile, our previous studies investigated the molecular pathway involved in cavernosal fibrosis after CN injuries, and suggested that the ROCK1/LIMK2/Cofilin pathway could contribute to cavernosal fibrosis with loss of SM after CN injuries [17, 18, 23]. In accordance with these, a few recent studies have demonstrated that activated RhoA/ROCK1 or ROCK2 pathway contributes to the development of ED and structural alterations such as cavernosal apoptosis or fibrosis in a rat model of CN injury [19, 20, 29]. According to the study by Gratzke et al., an increased protein expression of RhoA and ROCK2 was observed together with a decrease in erectile response to electrostimulation in a rat model of CNCI [19]. Also, intracavernous injection of ROCK inhibitors induced a significantly greater increase of ICP in nerve injured rats compared to that in sham operated rats, suggesting increased Rho kinase activity [19]. Hannan et al. demonstrated that intracavernous injection of ROCK inhibitors improved ED and cavernosal apoptosis through decreased protein expression of ROCK2 and reduced ROCK activity [20]. Interestingly, Mahmood et al. showed that radiation-induced CN injury caused ED through an increase of cavernosal fibrosis and apoptosis in rats [29]. Activated RhoA/ROCK1 pathway was involved in the cavernosal fibrosis and apoptosis after radiation-induced CN injury [29].

Thus, a few studies including our previous study have shown that inhibition of ROCK1 or ROCK2 rectifies ED by suppression of cavernosal apoptosis or fibrosis in a rat model of CN injury [23]. However, ROCK inhibitors have potential side effects, such as systemic vasodilation or hypotension [30, 31]. Fasudil, a potent ROCK inhibitor, is used as a local applicant to improve and prevent cerebral vasospasm or cerebral ischemia after subarachnoid hemorrhage surgery in China and Japan [32]. Ripasudil, a ROCK inhibitor eye drops, is also used a local applicant for glaucoma and ocular hypertension in Japan [33]. However, these ROCK inhibitors have not yet been approved by the US and Europe because of the risk of systemic adverse effects. Therefore, inhibition of LIMK, a downstream effector of ROCK, would be better treatment option than targeting ROCK itself [34].

In the next step, our recent study showed that LIMK2 inhibition, particularly with daily administration of 10.0 mg/kg LIMK2 inhibitors, for 1 week beginning from the immediate post-injury period improved cavernosal fibrosis and erectile response to electrostimulation by normalizing the LIMK2/Cofilin pathway in a rat model of CNCI [22]. Thus, we identified the possibility of an early therapeutic strategy targeting the LIMK2/Cofilin pathway to alleviate post-RP ED through improvement of cavernosal fibrosis caused by CNCI. With respect to the pathophysiological basis of post-RP ED, the present study demonstrated that chronic administration of LIMK2 inhibitors alleviated CVOD through suppression of post-injury cavernosal fibrosis. Taken together, our study may extend the current understanding regarding post-RP ED associated with cavernosal fibrosis induced by CN injuries.

Fibroblasts and myofibroblasts have been identified as key fibrosis effectors in many organs, and they are responsible for the synthesis of extracellular matrix proteins [11]. A major conserved cellular element of fibrosis is the activated fibroblast, known as a myofibroblast, which produces abundant amounts of extracellular matrix [11]. ROCK1 phosphorylates the downstream effector, the LIMK2/Cofilin pathway, which leads to cytoskeletal rearrangements and then to fibroblast-to-myofibroblast differentiation, a pathophysiological feature of fibrosis [35]. An important thing that leads to failure of cavernosal SM to expand is the structural alteration such as cavernosal fibrosis [7]. Since a long time, alleviation of cavernosal fibrosis has been a dream to get a durable erection and to overcome CVOD. In the current study, the fibroblasts positive for phosphorylated Cofilin were distributed mostly around the subtunical area. Based on the results of our previous study and the present study [18, 22, 23], the proliferation of fibroblasts positive for phosphorylated Cofilin in the subtunical area appears to play a role in CVOD caused by cavernosal fibrosis after RP. Our study showed that chronic inhibition of the LIMK2/Cofilin pathway could improve CVOD by normalizing the amount of fibroblasts positive for phosphorylated Cofilin in the subtunical area. Thus, our study may contribute to elucidation of the role of LIMK2 inhibition in improvement of CVOD, the major pathophysiologic mechanism of post-RP ED. Similarly, a recent study using aged rats showed that the RhoA/ROCK1/LIMK2/Cofilin pathway was involved in cavernosal fibrosis and ED caused by advanced age, suggesting that these factors might be novel targets in the treatment of age-related cavernosal fibrosis [36].

Interestingly, our results showed that the content of α-SMA in the L group was slightly increased compared with that in the I group although the increment was only modest. This finding indicates that the LIMK2/Cofilin pathway might contribute partly to apoptosis or anti-proliferation of SM cells in the cavernosal tissue after CN injuries. In line with our finding, a previous in-vitro study using human colon tumor cells showed that the DAPK/LIMK/p-Cofilin complex participates in TNF-induced apoptosis [37]. However, because there is scarcity of data about contribution of the LIMK2/Cofilin pathway to apoptosis or anti-proliferation of SM cells particularly in cavernosal tissue, whether the LIMK2/Cofilin pathway plays a role, albeit partly, in cavernosal apoptosis remains to be determined.

Although chronic inhibition of LIMK2/Cofilin significantly improved the SM/collagen ratio, deposition of Collagen-1 or Fibronectin in the cavernosal tissue, and CVOD, it did not completely restore them to normal values. Given that chronic inhibition of LIMK2/Cofilin normalized the amount of fibroblasts positive for phosphorylated Cofilin, our findings confirmed that other structural alterations such as cavernosal apoptosis could play a role in the development of CVOD after CN injury. Also, given that chronic administration of LIMK2 inhibitors did not completely restore the SM/collagen ratio or the protein expression of Collagen-1 or Fibronectin to normal values, it can be suggested that other molecular pathways related to fibrosis could contribute to cavernosal fibrosis induced by CN injury. Therefore, subsequent studies are necessary to identify whether CVOD induced by CN injury can be normalized to the level of control values through a combination therapy of LIMK2 inhibitors and anti-apoptotic agents or other agents targeting other fibrotic pathways. Another limitation of the present study was that our analysis of the fibrotic pathway focused exclusively on ROCK1 without evaluating ROCK2. Therefore, future studies are needed to include ROCK2 regarding analysis of the molecular pathway.

Nevertheless, our results suggest that the LIMK2/Cofilin pathway can contribute to the development of post-RP CVOD by promoting cavernosal fibrosis. From a clinical viewpoint, our findings indicate that LIMK2 inhibition may alleviate post-RP ED, albeit incompletely, by improvement of CVOD via suppression of cavernosal fibrosis.

## Conclusions

On the basis of our data, chronic inhibition of LIMK2 may alleviate CVOD, and thereby post-RP ED by improving cavernosal fibrosis via normalizing the LIMK2/Cofilin pathway, although the recovery is incomplete. Thus, interventions targeting the LIMK2/Cofilin pathway, at least as a part of combination therapies, may be reasonable therapeutic options for alleviation of cavernosal fibrosis and CVOD induced by CN injury, leading to maximization of the chance of improvement in post-RP ED. In the future, further investigations are necessary to validate this therapeutic strategy.
